# Functional Impact of Sublobar Resection for Early Stage Lung Cancers

**DOI:** 10.3390/cancers18101632

**Published:** 2026-05-19

**Authors:** Francesco Petrella, Stefania Rizzo

**Affiliations:** 1 Department of Thoracic Surgery, Fondazione IRCCS San Gerardo dei Tintori, 20900 Monza, MB, Italy; 2 Department of Oncology and Hemato-Oncology, University of Milan, Via Festa del Perdono 7, 20122 Milan, MI, Italy; 3 Clinic of Radiology, Institute of Integrated Diagnostics of Southern Switzerland, Via Tesserete 46, 6900 Lugano, CH, Switzerland; 4 Faculty of Biomedical Sciences, Università della Svizzera Italiana (USI), Via G.Buffi 13, 6900 Lugano, CH, Switzerland

**Keywords:** non-small-cell lung cancer, segmentectomy, wedge resection, sublobar resection, quality of life, spirometry

## Abstract

**Background:** The increased detection of small peripheral non-small-cell lung cancers through screening programs has renewed interest in parenchyma-sparing sublobar resection as an alternative to lobectomy. While oncologic non-inferiority has been established, the functional impact of sublobar resection remains a key consideration. **Methods:** We reviewed evidence from landmark randomized controlled trials, recent meta-analyses, observational studies, and updated clinical practice guidelines. **Results:** In the CALGB 140503 trial, sublobar resection demonstrated only a modest 2-percentage-point advantage in preserved FEV1 and FVC at 6 months compared with lobectomy, a difference considered clinically marginal in patients with normal baseline pulmonary function. A meta-analysis of five randomized controlled trials confirmed that sublobar resection was associated with significantly less reduction in postoperative lung function. A retrospective study demonstrated that segmentectomy preserved FEV1 at 84.2% of preoperative values versus 69.9% after lobectomy at one year, with particular benefit observed in elderly patients and those with COPD. Volumetric analyses showed greater contralateral compensatory lung expansion after lobectomy, partially offsetting functional differences. Notably, patient-reported outcomes, including physical function, dyspnea, and cough scores, showed no significant differences between groups up to two years postoperatively. The ERS/ESTS 2025 guideline noted that segmentectomy is associated with reduced long-term dyspnea deterioration and may improve patients’ ability to tolerate subsequent treatments. **Conclusions:** Sublobar resection offers a statistically significant but modest advantage in spirometric lung function preservation over lobectomy for early-stage NSCLC. This benefit may be most clinically relevant in patients with compromised baseline pulmonary function, COPD, or a potential need for future treatments.

## Simple Summary

Parenchyma-sparing sublobar resection has gained renewed interest for the treatment of small peripheral non-small-cell lung cancers detected through screening. Although oncologic outcomes are comparable to those of lobectomy, the functional benefits remain modest. Randomized trials have shown only small improvements in postoperative lung function, with limited clinical relevance in patients with normal baseline pulmonary status. Meta-analyses and observational studies suggest greater preservation of FEV1, particularly in elderly patients and those with COPD. However, compensatory lung expansion after lobectomy reduces these differences, and patient-reported outcomes appear similar between the two approaches. Overall, sublobar resection offers modest functional advantages, which are most relevant for patients with impaired pulmonary reserve or an anticipated need for future treatments.

## 1. Introduction

For nearly three decades after the 1995 Lung Cancer Study Group (LCSG) trial, lobectomy remained the established standard surgical treatment for patients with clinical T1N0 non-small-cell lung cancer (NSCLC) [1]. This landmark study reported a threefold increase in local recurrence and a 50% higher rate of lung cancer-related mortality following sublobar resection, thereby discouraging parenchyma-sparing approaches in operable patients [1].

However, improvements in imaging techniques, staging accuracy, and the widespread adoption of lung cancer screening programs have facilitated the detection of smaller, earlier-stage tumors. These developments have renewed interest in sublobar resections—namely segmentectomy and wedge resection—as potentially oncologically sound alternatives to lobectomy in carefully selected patients [2].

Three key randomized controlled trials have significantly reshaped this field. The JCOG0802/WJOG4607L trial demonstrated that anatomic segmentectomy was not only non-inferior but also superior to lobectomy in terms of overall survival (5-year OS: 94.3% vs. 91.1%) in patients with peripheral tumors ≤ 2 cm. However, locoregional recurrence was approximately twice as high in the segmentectomy group at 5-year follow-up [3]. Notably, this survival advantage was not sustained at 10-year follow-up [4], although methodological and statistical considerations have been proposed to explain this discrepancy [5].

The CALGB 140503 trial, an international phase III non-inferiority study involving 697 patients, further demonstrated that sublobar resection (including both wedge resection and segmentectomy) was non-inferior to lobectomy in terms of disease-free survival (HR 1.01; 90% CI, 0.83–1.24), with comparable overall survival outcomes (5-year OS: 80.3% vs. 78.9%) after a median follow-up of 7 years [6]. Similar findings were reported in a European randomized trial conducted by Stamatis et al. (DRKS00004897) [7].

Collectively, these results have driven a paradigm shift that is now reflected in updated clinical guidelines from the European Respiratory Society/European Society of Thoracic Surgeons (ERS/ESTS) [8], the American College of Chest Physicians (ACCP) [9], and the NCCN (2026) [10], all of which recognize sublobar resection as an equivalent alternative for patients with peripheral, node-negative tumors ≤ 2 cm.

One of the primary theoretical advantages of sublobar resection is the preservation of pulmonary function through parenchymal sparing. Nevertheless, randomized evidence suggests that this benefit may be modest. In the CALGB 140503 trial, the difference in median percent-predicted FEV1 at 6 months postoperatively was only 2 percentage points in favor of sublobar resection [6]. Similarly, the ACCP guidelines described the difference in postoperative pulmonary function between the two approaches as “quite small” [9].

In contrast, retrospective studies have reported more substantial functional differences. A large single-center study involving 1284 patients found that single segmentectomy preserved FEV1 at 84.2% of preoperative values at 12 months, compared with 69.9% after lobectomy, while multiple segmentectomies showed intermediate outcomes [11].

The ERS/ESTS 2025 guidelines acknowledged that sublobar resection provides a “variable degree of functional preservation,” while emphasizing that most available data derive from patients with relatively preserved baseline pulmonary function. Consequently, the clinical benefit may be more pronounced in individuals with limited respiratory reserve [8].

This review aims to evaluate the most recent evidence regarding the functional impact of sublobar resection in early-stage lung cancer, with particular attention to its clinical implications and oncologic outcomes in this patient population.

## 2. Search Strategy

The following narrative review examines the principal aspects of the functional consequences of sublobar resection in patients with early-stage lung cancer. Studies were selected according to their clinical relevance, methodological rigor, publication recency, and relevance to postoperative functional outcomes following lung resection. A comprehensive search of the literature was conducted using PubMed, MEDLINE, and Google Scholar to identify pertinent publications. The search strategy included the following MeSH terms: “non-small cell lung cancer”, “segmentectomy”, “wedge resection”, “sublobar resection”, “quality of life”, and “spirometry.” Eligible articles were required to report patient demographics, clinical features, and management strategies relevant to the subject. Clinical trials, cohort studies, and case–control studies published in English up to April 2026 were considered for inclusion. Additionally, the reference lists of the selected studies were manually reviewed to identify other relevant publications. Letters to the editor, conference abstracts, and non-peer-reviewed preprints were excluded from the review.

In this paper, we define wedge resection as a non-anatomic lung parenchymal excision that removes the tumor with a surrounding cuff of normal lung without individually dissecting and dividing the segmental bronchovascular pedicle, whereas segmentectomy is an anatomic sublobar resection that removes one (or more) bronchopulmonary segment(s) by identifying/dividing the segmental artery(ies), vein(s), and bronchus and separating along the intersegmental plane.

## 3. Historical Background

On 5 April 1933, Evarts A. Graham performed the first successful lung cancer resection through pneumonectomy [12]. In 1939, Edward D. Churchill and Ronald Belsey performed the first pulmonary segmentectomy—a lingulectomy—for bronchiectasis [13,14].

More than two decades later, in 1962, Michael B. Shimkin evaluated outcomes of lobectomy versus pneumonectomy in patients with lung cancer using data from Alton Ochsner and Richard H. Overholt. Their findings suggested that less extensive resections achieved survival outcomes comparable to, or possibly better than, those of more radical procedures [15].

In 1973, Robert J. Jensik and colleagues reported a 15-year experience involving 119 selected patients who underwent 123 segmental resections, with a mortality rate of 4.8%. Indications for segmentectomy included prior contralateral lung resection, the need for palliative intervention, and removal of peripheral lesions. Among patients undergoing radical resection, the 5-year survival rate was 56.4% [16].

In 1994, William H. Warren and Lorenzo P. Faber from the same institution compared outcomes between segmentectomy and lobectomy in patients with stage I lung cancer. Their analysis demonstrated no survival difference for tumors measuring 3.0 cm or less, whereas lobectomy provided a survival advantage for tumors larger than 3.0 cm. In addition, local recurrence was significantly higher after segmentectomy (22.7%) than after lobectomy (4.9%). Based on these findings, lobectomy was recommended for stage I NSCLC tumors exceeding 3.0 cm, whereas no clear benefit was identified for smaller tumors [17].

In 1995, Robert J. Ginsberg and colleagues published the results of the LCSG 0821 randomized trial comparing sublobar resection with standard lobectomy. The study demonstrated that sublobar resection did not confer significant benefits in terms of postoperative morbidity, mortality, or long-term cardiopulmonary function. Furthermore, because of increased mortality and higher local recurrence rates, lobectomy was established as the preferred surgical approach for patients with peripherally located T1N0 NSCLC [1].

Subsequently, lobectomy remained the treatment of choice for fit patients with early-stage NSCLC, whereas sublobar resections were reserved primarily for individuals with compromised cardiopulmonary reserve.

More recently, between 2022 and 2023, randomized non-inferiority trials conducted by the Japanese Clinical Oncology Group, the Cancer and Leukemia Group B (CALGB; now part of the Alliance for Clinical Trials in Oncology), and the Deutsche Register Klinischer Studien compared lobectomy with sublobar resection in patients with T1aN0 tumors measuring up to 2 cm. Despite differences in study design, patient populations, and reported outcomes, all trials achieved their primary endpoints, demonstrating that sublobar resections are non-inferior to lobectomy in terms of overall and disease-free survival [18].

**Figure 1:**
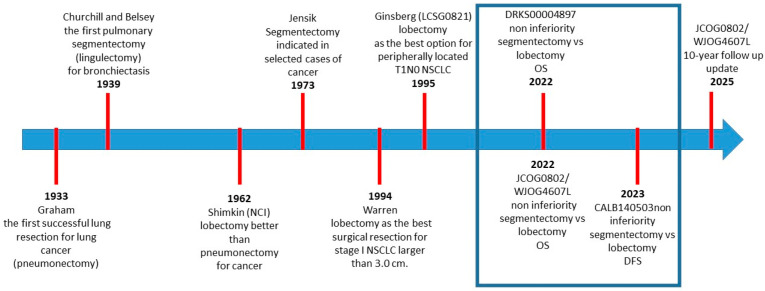
Timeline of the major milestones in the evolution of lung resection volumes.

Furthermore, a 10-year follow-up analysis of the JCOG0802/WJOG4607L trial confirmed the survival advantage of segmentectomy over lobectomy, demonstrating a slightly greater overall survival benefit at 10 years than that observed in the 5-year analysis. These findings reinforce earlier conclusions and support segmentectomy as a standard surgical option for this specific patient population [4,5] (Figure 1).

## 4. Pathophysiology of Pulmonary Function Changes After Lung Resection

The pathophysiology of pulmonary function changes after lung resection involves an immediate mechanical loss of lung tissue followed by compensatory mechanisms that vary according to baseline spirometric values and the extent of resection. The relationship between these factors is complex and reflects both anatomic reduction and physiologic adaptation.

FEV1 reaches its nadir immediately after surgery, with the magnitude of decline directly proportional to the extent of resection [19]. Following lobectomy, patients experience an initial decline, with FEV1 partially recovering by 6 months to a residual deficit of 9–11% from baseline. After pneumonectomy, recovery is markedly more limited, with FEV1 at 6 months ranging between 34–41% of preoperative values, and some studies reporting recovery to only 66% of baseline. Exercise capacity (VO_2_max) similarly decreases by 0–13% after lobectomy but remains 20–28% below baseline after pneumonectomy [19].

Patients with preexisting airflow limitation demonstrate paradoxically better postoperative outcomes than predicted by traditional calculations. Individuals with COPD often experience smaller reductions in postoperative expiratory volume than patients with normal spirometry, with an inverse relationship between actual postoperative FEV1 loss and baseline severity of airflow obstruction. This phenomenon may reflect lung volume reduction effects in hyperinflated patients, in whom resection of diseased tissue improves the mechanics of the remaining lung [19]. A preoperative FEV1 < 30% has been associated with respiratory morbidity rates as high as 43%, compared with 12% in patients with FEV1 > 60%. However, modern surgical techniques, including video-assisted thoracoscopic surgery (VATS), have enabled acceptable outcomes even in patients with markedly impaired baseline function [19].

The relationship between extent of resection and pulmonary function loss is non-linear and varies by procedure type [20]. In VATS procedures, pulmonary function loss averages approximately 5% per segment for lobectomy but approximately 10% per segment for segmentectomy. This apparent paradox occurs because lobectomy allows greater compensatory expansion of the remaining lobes, whereas segmentectomy may limit expansion due to residual intersegmental constraints [20].

Wedge resection best preserves pulmonary function, with changes similar to those observed after mediastinal procedures without lung resection [20]. Complex segmentectomy shows outcomes comparable to simple segmentectomy and significantly better preservation of lung function than lobectomy [21,22]. The number of resected segments is an independent risk factor for long-term decline in both FEV1 (OR 2.09) and vital capacity (OR 1.36) [23].

Recent evidence also demonstrates significant lobe-specific differences in recovery patterns [24]. Left upper and right lower lobectomies show observed pulmonary function values similar to predicted values as early as 2 weeks postoperatively, whereas resections of other lobes result in approximately 25% decline regardless of the number of segments removed. By 3 months, observed values exceed predicted values across all lobes, suggesting compensatory mechanisms such as hyperinflation of remaining lung tissue, improved ventilation–perfusion matching, and increased efficiency of respiratory muscles [24].

The consistent finding that actual postoperative function exceeds predicted values—particularly after 3 months—reflects multiple compensatory processes. Traditional segmental calculation methods underestimate postoperative FEV1 by approximately 250 mL after lobectomy and 500 mL after pneumonectomy [25]. This discrepancy is attributed to compensatory hyperinflation of the remaining lung, redistribution of ventilation and perfusion, and improved respiratory mechanics, particularly in patients with baseline airflow obstruction.

Long-term pulmonary function changes beyond 6 months remain insufficiently studied [19]. Postoperative complications and the number of resected segments are independent risk factors for persistent decline in vital capacity at 12 months, which negatively affects overall survival (HR 2.02) [23]. Patient-reported outcomes indicate that although objective pulmonary function improves, symptoms such as dyspnea and fatigue may persist beyond one year [26].

### 4.1. Functional Changes in Randomized Trials

JCOG0802/WJOG4607L trial

The JCOG0802/WJOG4607L trial was a multicenter, open-label, phase 3 randomized non-inferiority study conducted at 70 institutions in Japan. It enrolled 1106 patients with clinical stage IA NSCLC (tumor ≤ 2 cm, consolidation-to-tumor ratio > 0.5), who were randomized 1:1 to segmentectomy (n = 552) or lobectomy (n = 554) [3]. Postoperative respiratory function at 6 and 12 months was a prespecified secondary endpoint.

The primary spirometric parameter was forced expiratory volume in 1 s (FEV1), assessed as the percentage reduction from preoperative baseline. A ≥10% difference in median FEV1 reduction between groups was prespecified as the threshold for a clinically meaningful functional advantage of segmentectomy.

Eligibility criteria required an expected postoperative FEV1 of ≥800 mL and a partial pressure of arterial oxygen ≥ 65 Torr, ensuring adequate baseline pulmonary reserve in enrolled patients.

Both surgical approaches resulted in statistically significant declines in FEV1 from baseline (*p* < 0.0001 for both groups):

- Segmentectomy group
  o 6 months: median FEV1 reduction 10.4% (IQR 4.7–16.6);
  o 12 months: median reduction 8.5% (IQR 3.5–14.8).
- Lobectomy group
  o 6 months: median FEV1 reduction 13.1% (IQR 7.0–20.5);
  o 12 months: median reduction 12.0% (IQR 5.6–18.8).

Both groups showed partial recovery in FEV1 between 6 and 12 months. The segmentectomy group improved from a 10.4% to an 8.5% reduction, while the lobectomy group improved from 13.1% to 12.0%. This pattern suggests ongoing compensatory lung adaptation, although the magnitude of recovery was modest.

The between-group differences in median FEV1 reduction were:

- 6 months: 2.7% in favor of segmentectomy;
- 12 months: 3.5% in favor of segmentectomy (*p* < 0.0001).

Although statistically significant, the 3.5% difference at 12 months did not reach the prespecified 10% threshold defined as clinically meaningful. Thus, the functional benefit of segmentectomy was substantially smaller than originally hypothesized.

Several mechanisms likely explain this finding:

Compensatory lung expansion: After lobectomy, the remaining ipsilateral and contralateral lung undergoes compensatory hyperinflation and volume expansion, partially offsetting functional loss. Contralateral lung expansion is greater after lobectomy than after segmentectomy, narrowing the functional difference [27].

Intersegmental plane effects: Segmentectomy requires creation of intersegmental planes, which may lead to localized atelectasis, scarring, and distortion of adjacent segments, partially offsetting the expected parenchyma-sparing benefit. Functional outcomes vary by segment and by the number of intersegmental planes created [28].

Patient selection: The trial enrolled patients with relatively preserved baseline pulmonary function. The American College of Chest Physicians (ACCP) 2025 guideline and the CALGB 140503 investigators have noted that the functional benefit of sublobar resection is likely more clinically relevant in patients with impaired baseline pulmonary function or COPD, who were underrepresented in these trials [6,9].

Despite the modest spirometric difference, the trial demonstrated a significant overall survival advantage for segmentectomy (5-year OS 94.3% vs. 91.1%; HR 0.663, *p* = 0.0082 for superiority) [3]. This benefit has been partly attributed to better preservation of pulmonary reserve, enabling more aggressive treatment of recurrences and second primary lung cancers.

In the segmentectomy group, 93% of patients with recurrence received intensive treatment (including reoperation in 13 patients, radiotherapy in 13, chemotherapy in 32, and chemoradiotherapy in 4), compared with 80% in the lobectomy group. Similarly, 89% of patients with second primary lung cancers in the segmentectomy group underwent additional surgical resection, compared with 63% in the lobectomy group.

These findings suggest that even modest preservation of pulmonary function may translate into meaningful downstream clinical benefits by maintaining eligibility for subsequent curative interventions.

Finally, the ERS/ESTS 2025 guideline notes that segmentectomy is associated with reduced long-term deterioration in patient-reported dyspnea, suggesting a quality-of-life benefit that may not be fully captured by spirometric measures alone [8] (Table 1).

### 4.2. CALGB 140503 Trial

The CALGB 140503 trial, a landmark phase 3 non-inferiority study, randomized 697 patients with peripheral cT1aN0 NSCLC (≤2 cm) to sublobar resection (SLR, n = 340) versus lobar resection (LR, n = 357). Pulmonary function was a prespecified secondary endpoint.

At 6 months postoperatively, the trial demonstrated a statistically significant but clinically modest advantage of sublobar resection over lobectomy in preserving expiratory flow rates:

- FEV1 (% predicted): median reduction from baseline was −4.0 (95% CI, −5.0 to −2.0) after sublobar resection versus −6.0 (95% CI, −8.0 to −5.0) after lobar resection, corresponding to a 2-percentage-point difference favoring sublobar resection.
- FVC (% predicted): median reduction was −3.0 (95% CI, −4.0 to −1.0) after sublobar resection versus −5.0 (95% CI, −7.0 to −3.0) after lobar resection, again a 2-percentage-point difference favoring sublobar resection [6].

In a post hoc analysis comparing the three surgical approaches (lobectomy, segmentectomy, and wedge resection), the median reduction in %FEV1 at 6 months was 5% after wedge resection and 3% after segmentectomy (*p* = 0.930), indicating no significant difference between the two sublobar techniques [29].

The CALGB 140503 investigators acknowledged that the observed 2-percentage-point absolute difference in FEV1 and FVC between sublobar resection and lobar resection is “arguably not clinically meaningful” in patients with normal baseline pulmonary function. However, they highlighted several important considerations:

(a) Compromised patients: The functional advantage of sublobar resection may be more clinically relevant in patients with pre-existing airflow limitation (e.g., COPD) or reduced pulmonary reserve, where even small absolute differences in FEV1 may translate into meaningful symptomatic or functional effects.
(b) Lower-lobe disease: Lobectomy for lower-lobe tumors may be associated with greater impairment of pulmonary function due to the larger volume of resected parenchyma and the disproportionate contribution of lower lobes to ventilation.
(c) Single time-point measurement: A single spirometric assessment at 6 months may not fully capture the trajectory of pulmonary function recovery, which may continue to evolve over 12–18 months.
(d) Limitations of spirometry: Spirometry alone may underestimate the true functional impact of resection. The investigators suggested that measures such as the 6-min walk test or cardiopulmonary exercise testing (CPET) may better capture functional preservation.

The 2025 ERS/ESTS guideline echoed these concerns, noting that sublobar resections provide a “variable degree of functional preservation” as assessed by spirometry, and that subjective measures such as dyspnea scores and patient-reported outcomes may represent more clinically meaningful endpoints [8].

Overall, the CALGB 140503 spirometric data demonstrate a statistically significant but small absolute functional advantage for sublobar resection in a population with largely normal baseline lung function. This finding should be interpreted in the context of robust compensatory mechanisms that mitigate functional loss after lung resection. The clinical relevance of this difference is likely greater in patients with compromised pulmonary reserve, in whom sublobar resection may help preserve the functional threshold necessary to avoid respiratory disability and maintain eligibility for subsequent treatments for recurrence or second primary lung cancers [Table 2].

The DRKS00004897 Trial

The DRKS00004897 trial was a prospective, randomized, multicenter phase III study conducted across European centers, comparing anatomic segmentectomy versus standard lobectomy for pathological stage IA NSCLC (tumors ≤ 2 cm) [7]. Between October 2013 and June 2016, 108 patients with verified or suspected NSCLC ≤ 2 cm were enrolled and randomized 1:1 to segmentectomy (n = 53 completing) or lobectomy (n = 54).

The primary endpoint was 5-year overall survival (OS), with quality of life and pulmonary function preservation as secondary endpoints. At 5-year follow-up, OS was 86.5% in the lobectomy group versus 78.2% in the segmentectomy group (HR 0.61, 95% CI 0.23–1.66). Non-inferiority of segmentectomy for OS could not be formally demonstrated (p-ni = 0.687), although disease-free survival was non-inferior (77.3% vs. 78.0%, p-ni = 0.019). Rates of locoregional and distant recurrence were similar between groups (9.4% vs. 7.4%).

The trial was underpowered, enrolling only 108 patients, which limits the robustness of definitive conclusions [7].

Unfortunately, postoperative spirometric data were not reported in the available literature. The only available functional data relate to preoperative assessment, which showed comparable baseline values between groups:

- FEV1 (L): 2.29 (0.98–3.70) in the segmentectomy group vs. 2.25 (1.2–3.8) in the lobectomy group.
- FEV1 (% predicted): 82.1 (36.5–118.9) vs. 82.8 (32.7–132.0).
- DLCO (mL/mmHg/min): 66.55 (29.6–112.6) vs. 70.36 (34.6–104).
- Vital capacity (L): 3.30 (2.08–4.69) vs. 3.16 (2.41–4.69).

Overall, baseline pulmonary function was well balanced between the two groups [7] (Table 3).

## 5. Discussion

The physiological consequences of lung resection depend on the extent of parenchyma removed and involve interrelated changes in ventilatory mechanics, gas exchange, and exercise capacity. Following lobectomy, FEV1 reaches its nadir immediately postoperatively and then partially recovers. Importantly, postoperative pulmonary function recovery varies significantly according to the resected lobe.

A prospective longitudinal study found that left upper and right lower lobectomies demonstrated observed pulmonary function values similar to predicted postoperative (PPO) values as early as 2 weeks, whereas resections of other lobes showed an approximately 25% reduction in FVC, FEV1, and DLCO, regardless of the number of segments removed. By 3 months, observed values exceeded PPO values across all lobes, reflecting compensatory mechanisms [24].

The remaining lung undergoes compensatory adaptation through three main mechanisms: (1) recruitment of alveolar-capillary reserves, (2) remodeling of existing tissue, and (3) regenerative growth of acinar tissue when mechanical strain exceeds a critical threshold [30]. Experimental studies in canine models have demonstrated that two mechanical stimuli contribute approximately equally to this process: parenchymal stress from lung expansion and microvascular distension/shear stress from increased perfusion [31].

Compensatory growth is most pronounced after approximately 58% resection; beyond ~70% resection, further gains diminish, possibly due to excessive mechanical stress compromising septal integrity [32]. Importantly, while acinar structures regenerate effectively, conducting airways and vascular structures adapt more slowly. This mismatch may lead to disproportionate ventilatory and hemodynamic dysfunction and secondary right ventricular and respiratory muscle hypertrophy [30].

Lung resection also induces ventilation–perfusion (V/Q) mismatch, affecting gas exchange efficiency. Dynamic perfusion digital radiography studies have shown that pulmonary blood flow on the operated side reaches its nadir at 1 month postoperatively and then gradually recovers, with recovery patterns varying by resected lobe [33]. The interaction between functional lung volume recovery and perfusion redistribution is a key determinant of postoperative gas exchange efficiency.

A meta-analysis of 13 studies (2027 patients) confirmed that segmentectomy is associated with significantly better preservation of FEV1 and %FEV1 compared with lobectomy [34]. However, the magnitude of this difference in randomized trials has been modest. In CALGB 140503, the between-group difference at 6 months was only 2 percentage points for both FEV1 and FVC [6], while in JCOG0802, the difference in FEV1 reduction at 12 months was 3.5% [3].

By contrast, a retrospective study reported more pronounced differences at 1 year (FEV1 preservation of 84.2% vs. 69.9% of preoperative values for segmentectomy vs. lobectomy), although this was a non-randomized cohort [35]. The 2025 ACCP guideline noted that the small spirometric differences observed were particularly notable given that CALGB 140503 included a substantial proportion of wedge resections in the sublobar arm, a procedure involving even less parenchymal loss than segmentectomy [9].

The 2025 ERS/ESTS guideline emphasized that functional preservation likely varies according to the specific type of anatomical segmentectomy performed, and that bi- and multi-segment resections introduce additional heterogeneity in outcomes [8].

While spirometric differences appear modest, patient-reported outcomes provide a more nuanced perspective. Brunelli et al. reported that among long-term survivors (1–5 years postoperatively), 70% of lobectomy patients reported current dyspnea compared with 53% after segmentectomy (*p* = 0.035), and 82% reported perioperative dyspnea worsening versus 57% after segmentectomy (*p* = 0.002). After adjustment, segmentectomy was associated with a significantly reduced risk of dyspnea deterioration (OR 0.31, *p* = 0.004) [36].

Similarly, Stamatis et al.’s randomized trial showed that at 12 months, lobectomy was associated with greater deterioration in physical and cognitive function, dyspnea, and fatigue compared with baseline, whereas dyspnea recovered more rapidly after segmentectomy (*p* = 0.016) [7]. However, other studies have reported no significant differences in physical function, dyspnea, or cough between lobar and sublobar resection groups from 3 months to 2 years postoperatively [37]. Likewise, one study found that wedge resection did not significantly affect overall quality of life, whereas lobectomy was associated with significant declines in FACT-L scores driven by pain, fatigue, appetite loss, depression, and dyspnea [38].

A particularly important finding from the JCOG0802 trial was that the overall survival advantage of segmentectomy was not driven by a reduction in lung cancer-specific mortality, but rather by fewer deaths from other causes. In the overall cohort, 63% of deaths after lobectomy (52 of 83) were due to other diseases, compared with 47% after segmentectomy (27 of 58) [3].

The ERS/ESTS guideline further highlighted that patients undergoing sublobar resection may better tolerate subsequent treatments, which may contribute to improved long-term outcomes [8]. This is particularly relevant in the era of lung cancer screening, where patients frequently present with multiple lung nodules requiring sequential interventions, making parenchymal preservation a strategic consideration.

Finally, the robotic platform may be particularly advantageous in anatomic segmentectomy, where precise intersegmental plane identification and complex hilar dissection are required [39,40]. Echavarria et al. demonstrated that robotic segmentectomy is associated with significantly less predicted FEV1 and DLCO loss compared with robotic lobectomy (*p* < 0.001), confirming that the parenchyma-sparing benefit of segmentectomy is preserved in minimally invasive robotic surgery [41].

## 6. Limitations of Randomized Trials in Assessing Functional Outcomes

The key limitations of these trials in assessing respiratory functional outcomes can be summarized as follows:

(1) Reliance on FEV1 alone, without systematic inclusion of DLCO, cardiopulmonary exercise testing, or patient-reported outcomes as formal endpoints;
(2) Assessment at only one or two postoperative time points, which may not capture the full long-term trajectory of functional recovery;
(3) Inclusion of predominantly physiologically fit patients, limiting generalizability to individuals with impaired baseline pulmonary function who may derive the greatest benefit from parenchyma-sparing strategies;
(4) Insufficient accounting for heterogeneity among segmentectomy techniques and lobe-specific differences in functional recovery;
(5) In CALGB 140503, pooling of wedge resection and segmentectomy may obscure procedure-specific differences in functional preservation.

Collectively, these limitations suggest that the available randomized trials may have underestimated the functional benefit of sublobar resection, particularly in patient populations with reduced pulmonary reserve in whom such differences are most clinically relevant.

## 7. Conclusions

The functional impact of sublobar resection for early-stage lung cancer is characterized by a modest advantage in spirometric preservation, a more meaningful advantage in patient-reported dyspnea and quality of life, and a potentially important advantage in preserving physiological reserve for subsequent treatments. Although sublobar resection may reveal clinically relevant differences in selected populations, current definitive evidence remains heterogeneous. The higher locoregional recurrence rate observed with sublobar resection does not translate into a survival disadvantage in this carefully selected population. The relatively small spirometric differences measured in these trials may underestimate the true functional benefit, particularly in patients with compromised baseline function, and patient-reported outcomes and functional exercise testing may better capture the clinical significance of parenchyma preservation.

## Figures and Tables

**Table 1:** Spirometric changes in the JCOG0802/WJOG4607L trial.

Parameter	Segmentectomy	Lobectomy	Between-Group Difference
Median FEV1 reduction at 6 months	10.4% (IQR 4.7–16.6)	13.1% (IQR 7.0–20.5)	2.7%
Median FEV1 reduction at 12 months	8.5% (IQR 3.5–14.8)	12.0% (IQR 5.6–18.8)	3.5%

**Table 2:** Spirometric changes in the CALGB 140503 trial.

Resection Type	FEV1 Deficit at 6 Months	FVC Deficit at 6 Months
Wedge resection	~4–5%	~3%
Segmentectomy	~3–5%	~3–5%
Lobectomy	~6–11%	~5–7%

**Table 3:** Comparison of spirometric changes and key inclusion criteria in the JCOG0802/WJOG4607L trial, the CALGB 140503 trial and the DRKS00004897 trial.

Trial	Population (Key Inclusion)	Spirometric Changes
CALGB/Alliance 140503	Peripheral NSCLC, cT1aN0, tumor ≤ 2 cm	FEV1 Deficit at 6 Months Wedge resection: 4–5% Segmentectomy: 3–5% Lobectomy: 6–11% FVC Deficit at 6 Months Wedge resection: 3% Segmentectomy: 3–5% Lobectomy: 5–7%
JCOG0802/WJOG4607L	stage IA NSCLC (tumor ≤ 2 cm, consolidation-to-tumor ratio > 0.5)	Median FEV1 reduction at 6 months: Segmentectomy: 10.4% Lobectomy: 13.1% Median FEV1 reduction at 12 months Segmentectomy: 8.5% Lobectomy: 12.0%
DRKS00004897	Stage IA NSCLC (tumors ≤ 2 cm)	Not available in provided sources

## Data Availability

No new data were created or analyzed in this study. Data sharing is not applicable to this article.

## Abbreviations

COPD	Chronic obstructive pulmonary disease
FEV1	Forced Expiratory Volume in the 1st second
ERS	European Respiratory Society
ESTS	European Society of Thoracic Surgeons
RCT	Randomized Controlled Trial
NSCLC	Non-small-cell lung cancer
ACCP	American College of Chest Physicians
NCCN	National Comprehensive Cancer Network
OS	Overall Survival
DLCO	Diffusing Capacity of the Lung for Carbon Monoxide
FVC	Forced Vital Capacity
PPO	Predicted post-operative
FACT L score	Functional Assessment of Cancer Therapy—Lung
